# Screening of noise-induced hearing loss (NIHL)-associated SNPs and the assessment of its genetic susceptibility

**DOI:** 10.1186/s12940-019-0471-9

**Published:** 2019-04-04

**Authors:** Xuhui Zhang, Yaqin Ni, Yi Liu, Lei Zhang, Meibian Zhang, Xinyan Fang, Zhangping Yang, Qiang Wang, Hao Li, Yuyong Xia, Yimin Zhu

**Affiliations:** 1 Hangzhou Center for Disease Control and Prevention, Hangzhou, 310021 Zhejiang China; 2 Department of Epidemiology and Biostatistics, Department of Respiratory, Sir Run Run Shaw Hospital, Zhejiang University School of Medicine, Zhejiang Hangzhou, 310058 People’s Republic of China; 3 Hangzhou Hospital for Prevention and Treatment of Occupational Disease, Hangzhou, 310014 Zhejiang China; 4 Zhejiang Center for Disease Control and Prevention, Hangzhou, 310051 Zhejiang China; 5 Yongkang Center for Disease Control and Prevention, Yongkang, 321304 People’s Republic of China

**Keywords:** Genetic susceptibility, Noise-induced hearing loss (NIHL), Single nucleotide polymorphisms (SNPs), Genetic risk prediction, Genetic risk score (GRS)

## Abstract

**Background:**

The aim of this study was to screen for noise-induced hearing loss (NIHL)-associated single nucleotide polymorphisms (SNPs) and to construct genetic risk prediction models for NIHL in a Chinese population.

**Methods:**

Four hundred seventy-six subjects with NIHL and 476 matched controls were recruited from a cross-sectional survey on NIHL in China. A total of 83 candidate SNPs were genotyped using nanofluidic dynamic arrays on a Fluidigm platform. NIHL-associated SNPs were screened with a multiple logistic model, and a genetic risk model was constructed based on the genetic risk score (GRS). The results were validated using a prospective cohort population.

**Results:**

Seven SNPs in the *CDH23*, *PCDH15*, *EYA4*, *MYO1A*, *KCNMA1*, and *OTOG* genes were significantly (*P* < 0.05) associated with the risk of NIHL, whereas seven other SNPs were marginally (*P* > 0.05 and *P* < 0.1) associated with the risk of NIHL. A positive correlation was observed between GRS values and odds ratio (OR) for NIHL. Two SNPs, namely, rs212769 and rs7910544, were validated in the cohort study. Subjects with higher GRS (≧9) showed a higher risk of NIHL incidence with an OR of 2.00 (95% CI = 1.04, 3.86).

**Conclusions:**

Genetic susceptibility plays an important role in the incidence of NIHL. GRS values, which are based on NIHL-associated SNPs. GRS may be utilized in the evaluation of genetic risk for NIHL and in the determination of NIHL susceptibility.

**Electronic supplementary material:**

The online version of this article (10.1186/s12940-019-0471-9) contains supplementary material, which is available to authorized users.

## Introduction

Noise exposure is one of the most common occupational risk factors and have several detrimental effects on health, including irritability, insomnia, fatigue, and hearing loss [1]. Noise-induced hearing loss (NIHL) is a worldwide occupational health risk and the second most frequent form of sensorineural hearing loss, after age-related hearing impairment (ARHI) [2]. The World Health Organization (WHO) and the National Institute for Occupational Safety and Health (NIOSH) have classified NIHL as a disorder with a high priority for research (http://www.cdc.gov/NIOSH/).

NIHL is a complex disease that results from the interaction of occupational noise exposure, other risk factors like solvents, medication and vibration as well as life style factors (smoking and drinking status), and genetic risk factors [3, 4]. Noise exposure is associated with damage to the sensory cells of cochlea and the outer hair cells [5]. Oxidative stress and synaptic excitotoxicity are the major mechanisms of morphological pathologies [6]. However, under similar levels of noise exposure, workers may suffer different intensity of hearing damage. The differences indicate that genetic susceptibility plays an important role in the incidence of NIHL under noise-exposure environment. Previous animal and human studies have found that several genetic loci associated with the risk for NIHL [7]. Genetic variations in the *GSTM1*, *CAT*, *CDH23*, *KCNE1*, heat shock protein 70, and 8-oxoG DNA glycosylase 1 genes have been found to be associated with NIHL risk [8–13]. However, the genetic mechanism of NIHL pathogenesis still remains unclear. Most previous studies have focused on a few candidate SNPs, and the sizes of the study population were generally small [12–14].

In order to screen for NIHL-associated SNPs, we conducted a matched case–control study involving 476 NIHL workers and 476 normal hearing workers. We also established a genetic risk score (GRS) on the basis of these NIHL-associated SNPs and further examined these associations in a prospective cohort population.

## Methods

**Fig. 1:**
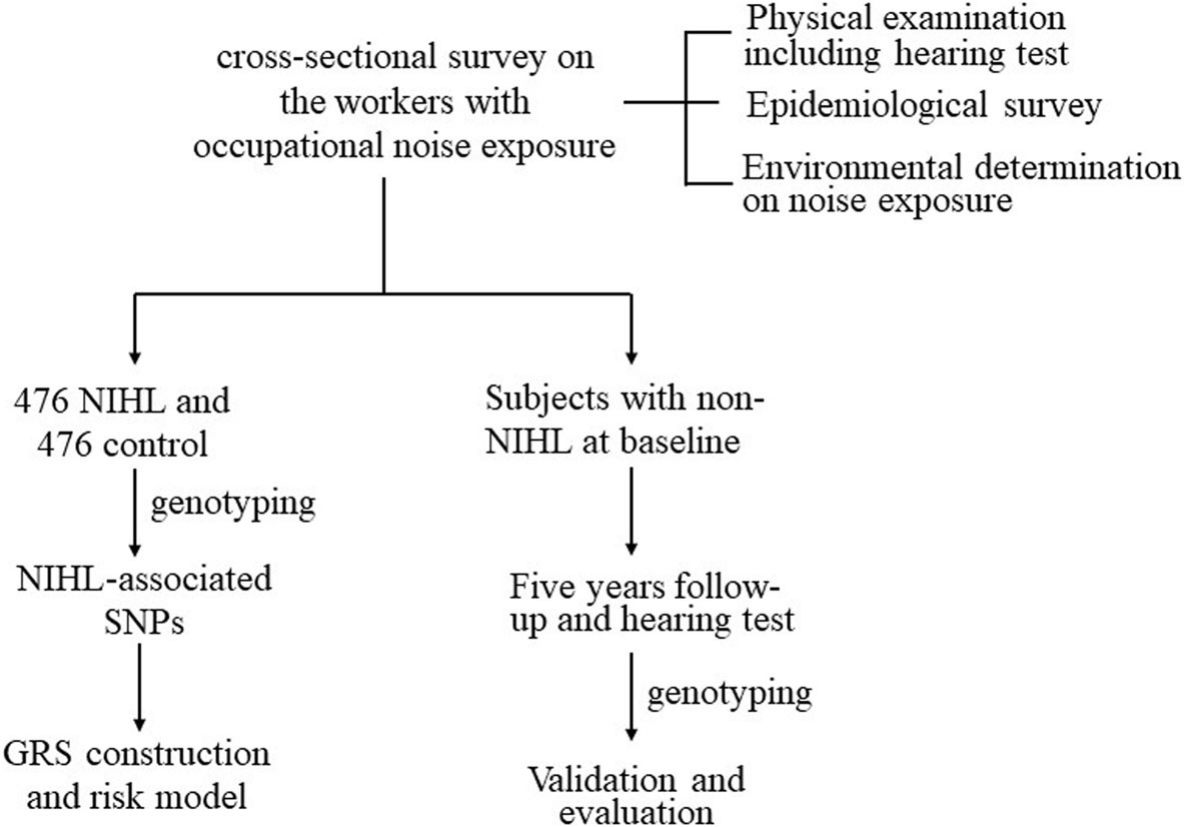
Flow chart of this study. The subjects were recruited form the cross-sectional survey on the workers with occupational noise exposure. Then 476 NIHL subjects and matched controls were genotyped. NIHL-associated SNPs were screened and GRS model was constructed. Four hundred eighty-five subjects with non-NIHL at baseline were further followed up and NIHL-associated SNPs and GRS model were further validated

NIHL-associated SNPs were screened from a case-control study and were validated in a prospective cohort. The flow chart of this study is presented in Fig. 1. The study protocol was approved by the Research Ethics Committees of Hangzhou Centers for Disease Prevention and Control, Zhejiang, China. All the participants provided the written informed consent.

### Cross-sectional survey of NIHL

A cross-sectional survey of occupational noise-exposed workers was conducted in 2011, and its procedure was previously described in detail [10, 11]. In this survey, environmental noise exposure was evaluated with equivalent continuous dB (A)-weighted sound pressure levels (LEX, 8 h) according to Occupational Health Standard of the People’s Republic of China: Measurement of Noise in the Workplace (GBZ/T189.8–2007) (China, 2007). All subjects who were exposed to occupational noise received annual health examinations, including routine physical examination, pure tone audiometry (PTA), epidemiological investigation, and whole blood collection. Hearing thresholds of both ears were determined with the ascending method in 5-dB steps at frequencies of 500 Hz, 1000 Hz, 2000 Hz, 3000 Hz, 4000 Hz, and 6000 Hz [10, 11].

The hearing threshold for each ear by PTA was the average at 3000 Hz, 4000 Hz, and 6000 Hz at high frequency (HTHF), and at 500 Hz, 1000 Hz, and 2000 Hz at speech frequency (HTSF). NIHL was defined using the following criteria: the workers with normal hearing before exposure, > 1 year of occupational noise exposure, and a HTHF > 40 dB. To exclude hearing loss was caused by factors other than noise, the worker was excluded from the study when the difference of HTHF between the left and right ears was > 35 dB (HL).

### Screening of NIHL-associated SNPs

To screen NIHL-associated SNPs, 476 NIHL male subjects, and gender-, intensity of noise-, and years of noise exposure-matched 476 controls were recruited from the subjects of a cross-sectional survey to screen for the NIHL- associated SNPs.

A total of 83 candidate SNPs in the 29 genes were selected as candidate SNPs in this study based on the HapMap database and previous reports [9–11, 15]. The inclusion criteria for searching for tag SNPs were as follows: minor allele frequency (MAF) in CHB > 0.05 and a linkage disequilibrium value of r2 > 0.8.

Whole blood was collected from each subject after an overnight fast. Genomic DNA was extracted from peripheral blood using the Toyobo MagExtractor Genomic DNA Purification Kit (Toyobo, Osaka, Japan) following the manufacturer’s protocol. All the candidate SNPs were genotyped using nanofluidic dynamic arrays on the Fluidigm platform (South San Francisco, CA, USA) at the Bio-X Institutes (Shanghai, China). Duplicate control samples were set in every genotyping plate, and the concordance was > 99%. SNPs were excluded when the call rates were < 90% and the controls deviated from Hardy-Weinberg equilibrium (HWE) with a *P* < 0.01.

### Follow-up and validation

Additional 584 subjects with normal hearing at baseline were further followed up after the cross-sectional study. Environmental noise exposure was monitored during this period using the same protocol with cross-sectional survey. After 5 years, the subjects were again administered a health check-up, including hearing threshold determination. The same definition of NIHL was used to assess the hearing status of the subjects after follow-up.

The SNPs, which were screened in the case-control study, were genotyped in the subjects of follow-up. The associations of the genotypes with the incidence of NIHL were examined.

### Statistics

Cumulative noise exposure (CNE) was calculated as CNE = 10 × log (10^SPL^ × years of noise exposure), where SPL is the sound pressure level [dB (A)] of noise exposure. Continuous variables for the normal distribution were expressed as the mean ± standard deviation (SD) and as the median (P25, P75) for skewed distribution. Categorical variables were expressed as frequency (%). Student’s *t*-test was used to examine the statistical significance of continuous variables, and the χ^2^ test for categorical variables. Hardy-Weinberg equilibrium (HWE) was tested using Pearson’s χ^2^ for each SNP in the control subjects, and the SNPs deviating from HWE were excluded from the analysis. A multiple logistic regression was used to calculate the odds ratio (OR) and 95% CI, modified by confounders such as age, smoking/drinking status, and CNE.

GRS was defined as the sum of the number of risk alleles (0, 1, 2 for additive model and 0, 1 for the dominant or recessive model, dependent of the most appropriate model) across NIHL-associated SNPs. GRS represents the overall risk of a specific disease after integrating a series of SNP markers and was calculated as the following formula [16, 17].

$$ \mathrm{GRS}=\sum \limits_{i=1}^k{n}_i $$

n_i_: the number of risk alleles in SNP_i_,

k: the number of NIHL-associated SNPs.

Considering the limited sample size and false negative signals, the SNPs, whose *P* values < 0.05 or marginal significance (0.05 < *P* < 0.10), were selected and used to construct the GRS model. The dose-response relationship between GRS and OR was tested using the χ^2^-square test for trends among the subjects with different levels of GRS.

All statistical analyses were performed using SPSS 19.0 for Windows (IBM Corporation, Armonk, NY, USA) and R software for Windows (3.3.1).

## Results

A total of 476 NIHL and 476 control subjects were recruited from the cross-sectional study for screening of NIHL-associated SNPs. However, one control subject failed to genotype, therefore, 475 controls were included in the final analysis. The basic characteristics of the subjects have been described in detail in our previous study [18]. Briefly, the mean age of the NIHL subjects was 36.6 ± 8.5 years (mean ± SD), significantly older than the control individuals (32.8 ± 8.0) (*P* < 0.001). There was no statistical significance between the NIHL subjects and controls regarding smoking, drinking status, years of noise exposure, and median of noise intensities (*P* > 0.05). CNE in the NIHL group was 95.5 (91.5, 100.5), and was slightly higher than the control group [94.3 (91.0, 97.8; *P* = 0.05).

Eighty-three SNPs in 29 genes were selected as candidate SNPs and genotyped in this study. Additional file 1: Table S1 describes the characteristics of these candidate SNPs and *P* values of association analysis under additive, dominant, and recessive models, respectively. After HWE testing, 11 SNPs were excluded from further analysis due to deviations from the equilibrium. Finally, seven SNPs in six genes were found to be significantly associated with NIHL (*P* < 0.05). The screened genes in the NIHL group included *CDH23*, *PCDH15*, eyes absent 4 homolog (*EYA4*), *MYO1A*, alpha subunit of calcium-activated potassium channel (*KCNMA1*), and *OTOG*. We also found that additional seven SNPs in six genes showed marginally significant association with NIHL (*P* > 0.05 and < 0.1).

Table 1 presents the ORs and 95% CI of these NIHL-associated SNPs. Besides rs11004085 in the *PCDH15* gene, rs3777781 and rs3777849 in *EYA4*, and rs2521768 in *DFNA5* that we have reported before [18, 19], additional 10 SNPs were found to be associated with NIHL risk. The variant alleles of rs1552245 in *MY01A*, rs4747192 in *CDH23*, rs1043421 in *MYO7A*, rs696211 in *KCNMA1*, and rs471757 in *GRHL2* decreased the risk for NIHL, whereas rs212769 in *EYA4*, rs2394795 in *CDH23*, rs7910544 in *KCNMA1*, rs3751385 in *CX43*, rs666026 in *GRHL2*, and rs7106021 in *OTOG* increased the risk for NIHL.

**Table 1:** Odd ratios (ORs) and 95% CI of NIHL– associated SNPs

Genotype	Control	NIHL	*P*-value^a^	OR (95% CI)^a^
Pcdh15/rs11004085				
TT	346	382	0.015	1
CT	120	88	0.005	0.59 (0.41, 0.85)
CC	5	3	0.395	0.53 (0.12, 2.31)
*P*_trend_			0.005	
CT/CC	125	91	0.004	0.59 (0.41, 0.84)
EYA4/ rs3777781				
TT	137	166	0.045	1
AT	236	235	0.116	0.76 (0.54, 1.07)
AA	96	71	0.015	0.57 (0.36, 0.90)
*P*_trend_			0.016	
AT/AA	332	306	0.047	0.72 (0.52, 0.99)
EYA4/ rs212769				
GG	354	336	0.094	1
AG	112	129	0.062	1.40 (0.98, 1.98)
AA	5	8	0.214	2.37 (0.61, 9.23)
*P*_trend_			0.033	
AG/AA	117	137	0.041	1.43 (1.01, 2.02)
MYO1A/rs1552245				
GG	293	308	0.088	1
AG	164	156	0.032	0.70 (0.51, 0.97)
AA	14	8	0.448	0.68 (0.25, 1.84)
*P*_trend_			0.032	
AG/AA	178	164	0.027	0.70 (0.51, 0.96)
DFNA5/ rs2521768				
TT	289	305	0.158	1
CT	155	148	0.249	0.82 (0.60, 1.14)
CC	27	19	0.089	0.52 (0.25, 1.10)
*P*_trend_			0.064	
CT/CC	182	167	0.122	0.78 (0.57, 1.07)
CDH23/rs2394795				
CC	134	108	0.122	1
TC	240	244	0.081	1.39 (0.96, 2.02)
TT	97	120	0.06	1.53 (0.98, 2.38)
*P*_trend_			0.058	
TC/TT	337	364	0.043	1.44 (1.01, 2.05)
CDH23/rs4747192				
CC	168	193	0.213	1
TC	230	220	0.113	0.77 (0.55, 1.06)
TT	73	60	0.189	0.73 (0.45, 1.17)
*P*_trend_			0.106	
TC/TT	303	280	0.084	0.76 (0.56, 1.04)
MYO7A/ rs1043421				
TT	252	282	0.16	1
AT	192	170	0.066	0.74 (0.54, 1.02)
AA	28	21	0.384	0.73 (0.37, 1.47)
*P*_trend_			0.071	
AT/AA	220	191	0.057	0.74 (0.55, 1.01)
KCNMA1/rs696211				
TT	247	247	0.052	1
CT	178	194	0.904	0.98 (0.71, 1.35)
CC	45	31	0.016	0.47 (0.26, 0.87)
*P*_trend_			0.080	
TT + CT	425	441	0.016	0.48 (0.26, 0.87)
KCNMA1/ rs7910544				
GG	343	345	0.175	1
CG	121	111	0.652	0.92(0.65, 1.31)
CC	7	16	0.077	2.70(0.90, 8.11)
*P*_trend_			0.576	
GG + CG	7	16	0.070	2.76(0.92,8.26)
CX43/rs3751385				
CC	131	109	0.134	1
CT	259	265	0.071	1.40 (0.97, 2.01)
TT	80	99	0.086	1.50 (0.94, 2.39)
*P*_trend_			0.081	
CT/TT	339	364	0.056	1.41 (0.99, 1.99)
GRHL2/ rs471757				
CC	158	167	0.161	1
CT	208	217	0.889	0.98 (0.69, 1.37)
TT	105	89	0.078	0.68 (0.45, 1.04)
*P*_trend_			0.104	
CC + CT	366	384	0.062	0.70 (0.48, 1.02)
GRHL2/ rs666026				
TT	244	222	0.147	1
GT	186	204	0.118	1.29 (0.94, 1.77)
GG	40	46	0.121	1.55 (0.89, 2.71)
*P*_trend_			0.051	
GT/GG	226	250	0.065	1.33 (0.98, 1.80)
OTOG/ rs7106021				
GG	340	326	0.031	1
GA	116	136	0.014	1.55(1.09, 2.20)
AA	14	10	0.485	0.72(0.29, 1.81)
*P*_trend_			0.123	
GA + AA	130	146	0.036	1.43(1.02, 2.00)

^a^ calculated with logistic regression adjusted by age, CNE, smoking, and drinking

**Fig. 2:**
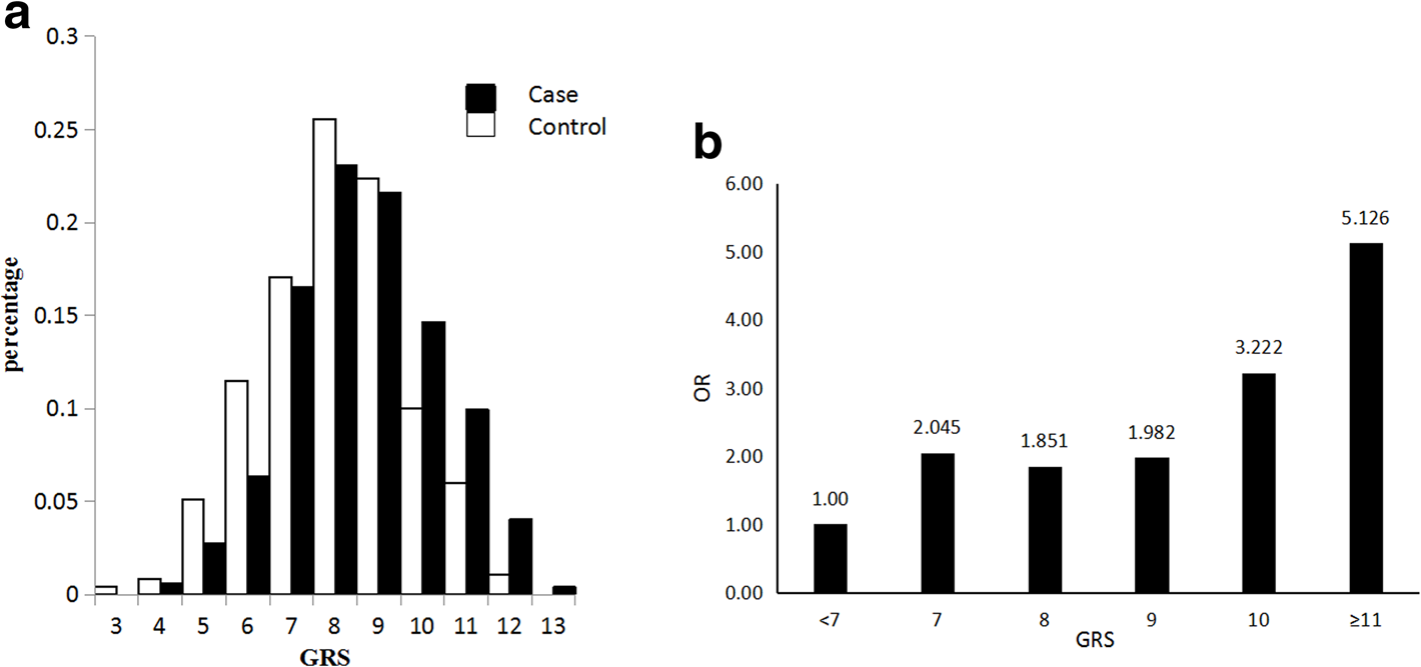
Distribution of GRS in the subjects of NIHL and controls. a, different distributions of GRS in the subjects with NIHL and control subjects. The distribution of GRS in the subjects with NIHL has right-tendency. b, dose-response relationship between the levels of GRS and risk of NIHL. The risk of NIHL positively correlates with the values of GRS

Different distributions of GRS were observed between individuals with NIHL and the controls (Table 2 and Fig. 2). The median (P_25_, P_75_) GRS of the NIHL group was 9.0 (7.0, 10.0), which was significantly higher than the controls (8.0 (7.0, 9.0)) (*P* value < 0.001). Figure 2-a also shows NIHL subjects have higher levels of GRS than those in control subjects. When the subjects were divided based on GRS levels, the ORs were calculated with the subjects with the lowest levels of GRS (< 7) as the reference group. The ORs significantly correlated to GRS values (*P*_trend_ < 0.01) (Table 2 and Fig. 2-b). If the subjects were further classified into two groups (high or low GRS) based on a GRS value of 9, then the subjects with GRS≧9 had increased risk for NIHL, with an OR of 1.58 (95% CI = 1.22, 2.04) compared to the subjects with GRS < 9.

**Table 2:** Associations between levels of GRS and risk for NIHL

GRS	Control	Case	Total	*P*-value	OR(95% CI)
Median (P25, P75)	8.0 (7.0, 9.0)	9.0 (7.0, 10.0)	8.0 (7.0, 9.0)	< 0.001	
GRS subgroup, n (%)					
< 7	84 (17.9)	46 (9.7)	130 (13.8)		1
7	80 (17.1)	78 (16.5)	158 (16.8)	0.005	2.04 (1.24, 3.38)
8	120 (25.6)	109 (23.1)	229 (24.3)	0.01	1.85 (1.16, 2.96)
9	105 (22.4)	102 (21.6)	207 (22.0)	0.005	1.98 (1.23, 3.20)
10	47 (10.0)	69 (14.6)	116 (12.3)	< 0.001	3.22 (1.86, 5.57)
≥11	33 (7.0)	68 (14.4)	101 (10.7)	< 0.001	5.13 (2.87, 9.16)
*P*_trend_				< 0.001	
< 9	284 (60.6)	233 (49.4)	517 (54.9)		1
≥9	185 (39.4)	239 (50.6)	424 (45.1)	0.001	1.58 (1.22, 2.04)

Five hundred eighty-four subjects, who had normal hearing capacity at baseline, were followed up in cohort. After 5 years, 51 subjects were assessed to have NIHL using the same criteria as baseline, and therefore, the overall incidence rate of NIHL was 8.85%. The incidence rates of the subjects with different genotypes are shown in Table 3. The genotypes of rs212769 in the EYA4 gene was associated with the risk for NIHL incidence (*P*_trends_ = 0.017). Compared to the subjects who were homozygous for allele (G), the subjects carrying the variant allele showed an increased risk for NIHL (OR: 6.71, 95% CI = 1.77–25.41) The subjects who were homozygous for the variant allele of rs7910544 in the KCNMA1 gene had an increased risk for NIHL (OR: 5.23, 95% CI = 1.20–22.71). No significant association was found in the other SNPs.

**Table 3:** Odd ratios (ORs) and 95% CI of NIHL– associated SNPs in the cohort study

Genotype or GRS	N	NIHL		*P*	OR (95% CI)
		n	%		
EYA4/ rs212769					
GG	440	36	8.18		1
AG	125	13	10.4	0.976	1.01(0.52, 2.19)
AA	11	4	36.36	0.001	28.27(4.21, 189.85)
AA vs GG/AG	11/565	4/49	36.36/8.67	0.005	6.710(1.77, 25.41)
KCNMA1/ rs7910544					
GG	424	46	10.85		1
CG	143	5	3.5	0.017	0.31(0.12, 0.81)
CC	9	2	22.22	0.157	3.72(0.60, 23.03)
CC vs CG/GG	9/567	2/51	22.22/8.99	0.027	5.23(1.20, 22.71)
GRS^a^					
< 7	46	2	4.35		1
7	75	4	5.33	0.537	1.74(0.30, 10.18)
8	105	7	6.67	0.425	1.97(0.37, 10.49)
9	129	15	11.63	0.084	3.99(0.83, 19.12)
≥10	211	22	10.43	0.163	2.92(0.65, 13.16)
*P*_trend_				0.092	
< 9	226	13	5.75		1
≥9	340	37	10.88	0.038	2.00(1.04,3.86)

^a^ *GRS* genetic risk score, was calculated as the sum of the risk allele in 14 NIHL-associated SNPs

The incidence rates of NIHL among subjects in different levels of GRS are also presented in Table 3. The incidence of NIHL was marginally correlated with GRS levels (P_trend_ = 0.092). Subjects with high GRS values (≥ 9) had a NIHL incidence of 10.88%, which is significantly higher than those with low GRS values (< 9) (OR = 2.00, 95% CI = (1.04, 3.86) (*P* = 0.038).

## Discussion

In this study, we screened 14 NIHL-associated SNPs in 10 genes in cross-sectional study. A genetic risk predictive model was constructed based on these SNPs. A dose–response relationship was found between the levels of GRS and NIHL risk. Two SNPs were also validated in the prospective cohort. These findings indicate that genetic susceptibility plays an important role in NIHL incidence. To the best of our knowledge, this is the first study that has been conducted to evaluate individual genetic susceptibility based on multiple SNP loci.

NIHL results from the interaction of occupational noise exposure and genetic risk factors. Due to the risk difference under similar noise exposure environments, it is seen that genetic susceptibility might play an important role in NIHL [3, 8]. Previous studies have uncovered several NIHL-associated SNPs in the *GSTM1*, *CAT*, *CDH23*, *KCNE1*, heat shock protein 70, and 8-oxoG DNA glycosylase 1 genes. In the Chinese population, we have identified that rs1104085 in the *PCDH15* gene, rs3777781 and rs212769 in the *EYA4* gene, rs666026 in the *GRHL2* gene, and rs2521768 in the *DFNA5* gene are associated with NIHL risk [10, 11]. However, the findings are still not enough to explain the incidence of hearing loss with noise exposure. In the present study, using the results of a case–control investigation, we had screened 14 NIHL-associated SNPs.

We previously reported that sequence variants in the *PCDH15*, *EYA4*, *GRHL2*, and *DFNA5* genes are associated with NIHL [10, 11]. Here, we also found that SNPs in the *CDH23*, *CX43*, *KCNMA1*, *MYO1A*, *MYO7A*, and *OTOG* genes are associated with NIHL. However, the effect of a single polymorphism locus is weak. Therefore, these genetic markers were integrated into an index, namely, GRS, to evaluate individual genetic predisposition to NIHL, similar to that performed on other specific complex diseases such as cancer, obesity, and diabetes [17, 20]. The NIHL group showed high GRS values compared to the controls, thereby indicating that subjects with NIHL have a higher genetic susceptibility than controls. We also found that subjects with high GRS values had a greater risk for NIHL (OR: 2.69, 95% CI = 1.71, 4.23) when compared with those with low GRS values. We also validated the GRS values of two SNPs, namely, rs212769 and rs7910544, in our prospective cohort. Using these genetic biomarkers, we were able to screen for NIHL-susceptible subjects, and discriminate higher sensitivity to NIHL from the noise-exposed workers. It is relatively difficult to avoid noise exposure under most occupational environments. Therefore, preventative measures for high-risk populations are essential. In this case, primary prevention (for etiological factors) is an effective and efficient measurement. Once screening and recognizing the susceptible individuals, we could take the measurements such as appropriate job selection, decreasing noise exposure, and strengthen protection (Putting on ear plugs or helmet) in noise environment in order to effectively reduce the risk of NIHL. Previous study had found that better use of hearing protection as part of a program probably helps but does not fully protect against hearing loss. Improved implementation might provide better protection [21].

The *EYA4* gene is a member of the vertebrate EYA family of transcriptional activators, and we previously conducted an investigation on this gene and found that The SNPs of rs3777781 and rs212769 in the *EYA4* gene were significantly associated with NIHL risk [10]. Furthermore, rs212769 also validated in the prospective study. The subjects with genotypes AA increased the risk of incidence of NIHL. This finding indicate that *EYA4* may play an important role in the incidence of NIHL. K^+^ is the main charge carrier of sound sensory conduction, and its normal circulation is important for the function of hearing. Previous studies have shown that sequence variants involving related genes may lead to hearing loss [1, 22–25]. Previous investigations on the associations between potassium circulation channel genes and NIHL have mainly focused on the *GJB2*, *GJB3*, *GJB6*, *KCNE1*, *KCNQ1*, and *KCNQ4* genes, and some have been validated in various populations [11]. KCNMA1 (BK channel), one of the potassium ion channel proteins, is expressed in the tubular system and renal vasculature, and mainly functions to control the transmembrane fluxes of K^+^ and Ca^2+^. However, KCNMA1 has been mainly studied in relation to tumorigenesis [26, 27]. In animal studies, mice lacking *KCNMA1* can develop normal hearing at early life, but then later show progressive hearing loss, indicating that *KCNMA1* is not essential for basic inner hair cell function [28, 29]. The potassium channels of KCNMA1 are apparently essential for the survival of outer hair cells, which are the major structural components of hearing [29, 30]. In this study, we found that the homozygote of mutant allele (CC) in rs696211 of KCNMA1 gene decreased and genotypes CC in rs7910544 increased the risk of NIHL. Therefore, the genetic variation of KCNMA1 modifies the risk of NIHL.

This may be the first study that has evaluated the individual genetic risk for NIHL under a noise-exposed environment based multiple SNP loci. One of the strengths of the study is that our preliminary established NHL risk prediction model using 14 SNPs to screen for High risk NIHL was partly validated in the follow-up study cohort using NIHL incidence over a 5 year period. However the sample size in the prospective cohort was relatively small and the follow-up time was relatively short, thus false negative results may still exist in this study. Further studies using both men and women as well as a larger sample size should be performed to validate the results.

Briefly, 14 SNPs in 10 genes were found to be associated with NIHL risk. Two SNPs, namely, rs212769 and rs7910544, were also validated in the prospective cohort study. GRS values for NIHL were significantly correlated with NIHL risk. These findings suggest that genetic susceptibility plays an important role in NIHL incidence, and thus provide a method of identifying individuals with higher genetic risk for NIHL.

## Additional file

Additional file 1: **Table S1**. Distribution of allele and genotype frequencies in the subjects of Case and Control. (DOCX 37 kb)

## Abbreviations

CI: Confidence interval
CNE: Cumulative noise exposure
GRS: Genetic risk score
MAF: Minor allele frequency
NIHL: Noise-induced hearing loss
OR: Odds ratio
SNP: Single-nucleotide polymorphism
