# Supplementary material for: Screening of noise-induced hearing loss (NIHL)-associated SNPs and the assessment of its genetic susceptibility
Source: Environ Health. 2019 Apr 4;18:30. doi: 10.1186/s12940-019-0471-9 (PMC6449917; doi:10.1186/s12940-019-0471-9)
Supplement: Supplementary file 1 — Table S1. Distribution of allele and genotype frequencies in the subjects of Case and Control. (DOCX 37 kb) [file 12940_2019_471_MOESM1_ESM.docx]

| Supplemental table S1. Distribution of allele and genotype frequencies in the subjects of Case and Control | | | | | | | | | | |
| --- | --- | --- | --- | --- | --- | --- | --- | --- | --- | --- |
| Gene | SNP | CHR | A1/A2 | MAF | *P*(H-W) | A1A1, A1A2, A2A2 | | *P*-  value^*^ | *P*-  Value# | *P*-  value§ |
|  |  |  |  |  |  | Case | Control |  |  |  |
| BDNF | rs7103411 | 11 | C/T | 0.5 | 0.23 | 111/249/113 | 111/249/111 | 0.495 | 0.752 | 0.444 |
| CDH23 | rs10823847 | 10 | T/C | 0.49 | 0.36 | 111/240/122 | 109/247/117 | 0.933 | 0.744 | 0.641 |
|  | rs1867977 | 10 | T/C | 0.45 | 0.26 | 94/240/139 | 89/246/136 | 0.711 | 0.561 | 0.973 |
|  | rs1867996 | 10 | T/G | 0.08 | 1 | 3/70/399 | 2/62/406 | 0.15 | 0.152 | 0.589 |
|  | rs1867998 | 10 | T/C | 0.21 | 0.78 | 20/155/297 | 21/151/298 | 0.339 | 0.304 | 0.752 |
|  | rs2394795 | 10 | T/C | 0.49 | 0.64 | 108/244/120 | 97/240/134 | 0.056 | 0.043 | 0.281 |
|  | rs3998530 | 10 | G/A | 0.28 | 0.64 | 39/197/237 | 33/193/245 | 0.886 | 0.833 | 0.493 |
|  | rs4747192 | 10 | T/C | 0.38 | 0.77 | 60/220/193 | 73/230/168 | 0.105 | 0.084 | 0.438 |
|  | rs6480521 | 10 | C/T | 0.38 | 1 | 75/220/177 | 62/217/192 | 0.271 | 0.174 | 0.776 |
|  | rs7093128 | 10 | T/C | 0.19 | 1 | 20/149/303 | 16/142/312 | 0.714 | 0.930 | 0.379 |
|  | rs748504 | 10 | A/G | 0.4 | 0.7 | 68/246/159 | 72/230/168 | 0.712 | 0.771 | 0.755 |
| CLDN14 | rs128494 | 21 | C/T | 0.48 | 0.46 | 103/238/132 | 105/243/122 | 0.262 | 0.118 | 0.816 |
| COCH | rs2295128 | 14 | C/A | 0.27 | 0.36 | 24/197/252 | 32/198/241 | 0.311 | 0.482 | 0.284 |
|  | rs3759777 | 14 | T/A | 0.49 | 0.31 | 114/235/124 | 110/247/114 | 0.842 | 0.764 | 0.515 |
| CX43 | rs3751385 | 13 | T/C | 0.47 | 0.02 | 99/265/109 | 80/259/131 | 0.085 | 0.055 | 0.448 |
| DFNA5 | rs2521758 | 7 | G/T | 0.02 | 0.22 | 0/14/458 | 1/20/450 | 0.039 | 0.052 | 0.999 |
|  | rs2521768 | 7 | C/T | 0.21 | 0.35 | 19/148/305 | 27/155/289 | 0.065 | 0.124 | 0.129 |
| DIAPH1 | rs2302103 | 5 | T/C | 0.31 | 0.92 | 38/203/231 | 47/202/221 | 0.137 | 0.250 | 0.170 |
|  | rs251018 | 5 | C/A | 0.16 | 0.6 | 10/143/320 | 13/122/336 | 0.815 | 0.784 | 0.981 |
| EYA4 | rs212769 | 6 | A/G | 0.14 | 0.31 | 8/129/336 | 5/112/354 | 0.033 | 0.042 | 0.292 |
|  | rs3777781 | 6 | A/T | 0.43 | 0.93 | 72/235/166 | 98/236/137 | 0.019 | 0.049 | 0.064 |
|  | rs3777849 | 6 | A/G | 0.35 | 0 | 70/175/228 | 85/182/204 | 0.042 | 0.149 | 0.037 |
|  | rs3777860 | 6 | A/G | 0.35 | 1 | 62/217/194 | 57/215/200 | 0.509 | 0.643 | 0.518 |
|  | rs465147 | 6 | T/C | 0.01 | 1 | 0/7/465 | 0/11/459 | 0.235 | 0.241 | - |
| Fign | rs2231899 | 2 | C/T | 0.4 | 0.11 | 76/222/175 | 71/246/154 | 0.702 | 0.182 | 0.332 |
| GRHL2 | rs10955255 | 8 | G/A | 0.22 | 0.61 | 16/164/292 | 23/173/275 | 0.499 | 0.475 | 0.865 |
|  | rs1981361 | 8 | A/G | 0.29 | 0.26 | 36/200/237 | 34/205/232 | 0.871 | 0.728 | 0.774 |
|  | rs471757 | 8 | T/C | 0.43 | 0.02 | 89/217/167 | 105/208/158 | 0.109 | 0.390 | 0.068 |
|  | rs666026 | 8 | G/T | 0.3 | 0.57 | 46/204/222 | 40/186/244 | 0.051 | 0.064 | 0.240 |
|  | rs682769 | 8 | A/G | 0.19 | 0.04 | 10/155/308 | 11/163/297 | 0.574 | 0.573 | 0.838 |
| Itga8 | rs10508489 | 10 | T/C | 0.01 | 1 | 0/10/463 | 0/15/457 | 0.165 | 0.166 | - |
| KCNMA1 | rs11595837 | 10 | T/C | 0.01 | 0 | 0/7/466 | 2/11/461 | 0.123 | 0.155 | 0.999 |
|  | rs1436089 | 10 | A/G | 0.01 | 1 | 0/8/464 | 0/7/463 | 0.843 | 0.847 | - |
|  | rs42311 | 10 | G/A | 0.18 | 0.04 | 7/137/328 | 11/164/296 | 0.185 | 0.197 | 0.496 |
|  | rs582273 | 10 | G/A | 0.04 | 1 | 0/36/437 | 0/40/431 | 0.549 | 0.532 | - |
|  | rs582449 | 10 | G/T | 0.25 | 0.15 | 31/167/275 | 25/194/253 | 0.524 | 0.344 | 0.757 |
|  | rs696211 | 10 | C/T | 0.28 | 0.14 | 31/194/247 | 45/178/247 | 0.082 | 0.372 | 0.016 |
|  | rs697171 | 10 | A/G | 0.11 | 0.82 | 1/96/376 | 5/96/371 | 0.381 | 0.573 | 0.998 |
|  | rs7083476 | 10 | A/C | 0.12 | 0.52 | 2/111/360 | 5/107/359 | 0.834 | 0.698 | 0.353 |
|  | rs7910544 | 10 | C/G | 0.15 | 0.45 | 16/111/345 | 7/121/343 | 0.576 | 0.989 | 0.070 |
|  | rs860989 | 10 | A/G | 0.2 | 0.77 | 16/154/303 | 20/149/301 | 0.906 | 0.994 | 0.749 |
|  | rs866865 | 10 | G/A | 0.25 | 0.01 | 28/171/274 | 44/162/265 | 0.326 | 0.576 | 0.217 |
| MYH14 | rs12608568 | 19 | A/C | 0.39 | 0.44 | 71/224/177 | 67/231/170 | 0.575 | 0.515 | 0.854 |
|  | rs3745504 | 19 | A/G | 0.35 | 0.69 | 52/214/206 | 64/212/192 | 0.962 | 0.663 | 0.461 |
|  | rs588035 | 19 | G/C | 0.07 | 0.71 | 5/67/401 | 1/60/408 | 0.197 | 0.212 | 0.556 |
| MYH9 | rs1557536 | 22 | G/C | 0.21 | 0.23 | 25/146/302 | 18/172/281 | 0.247 | 0.153 | 0.924 |
|  | rs5756129 | 22 | T/C | 0.28 | 0.41 | 35/199/237 | 30/193/246 | 0.973 | 0.912 | 0.881 |
| MYO1A | rs1552245 | 12 | A/G | 0.19 | 0.15 | 8/156/308 | 14/164/293 | 0.032 | 0.028 | 0.565 |
| MYO3A | rs11014993 | 10 | C/T | 0.27 | 0.48 | 28/194/251 | 30/191/250 | 0.598 | 0.656 | 0.720 |
| MYO6 | rs12213795 | 6 | G/A | 0.03 | 1 | 0/26/447 | 0/33/438 | 0.276 | 0.278 | - |
| MYO7A | rs1043421 | 11 | A/T | 0.24 | 0.34 | 21/170/282 | 28/192/252 | 0.068 | 0.055 | 0.518 |
| OTOA | rs215884 | 16 | A/T | 0.42 | 0.35 | 81/230/161 | 81/241/149 | 0.241 | 0.224 | 0.535 |
|  | rs741718 | 16 | C/T | 0.12 | 0.52 | 11/97/365 | 5/104/363 | 0.124 | 0.218 | 0.132 |
| OTOF | rs13004993 | 2 | A/G | 0.01 | 1 | 0/6/467 | 0/6/464 | 0.977 | 0.991 | - |
|  | rs4665327 | 2 | T/G | 0.03 | 1 | 0/20/452 | 0/29/442 | 0.273 | 0.274 | - |
| OTOG | rs7106021 | 11 | A/G | 0.16 | 0.29 | 10/136/326 | 14/116/340 | 0.123 | 0.036 | 0.339 |
| OTOR | rs6135876 | 20 | C/T | 0.3 | 0.02 | 48/196/229 | 53/176/242 | 0.893 | 0.531 | 0.462 |
| Pcdh15 | rs10509005 | 10 | G/A | 0.14 | 0.85 | 12/107/354 | 9/119/343 | 0.648 | 0.483 | 0.534 |
|  | rs10825122 | 10 | A/T | 0.15 | 0.73 | 10/122/341 | 10/127/334 | 0.222 | 0.161 | 0.815 |
|  | rs11004085 | 10 | C/T | 0.12 | 0.17 | 3/88/382 | 5/120/346 | 0.005 | 0.004 | 0.485 |
|  | rs11004270 | 10 | C/T | 0.17 | 0.1 | 12/138/323 | 8/142/321 | 0.577 | 0.300 | 0.172 |
|  | rs11004347 | 10 | A/G | 0.39 | 0.17 | 67/236/170 | 61/237/174 | 0.792 | 0.996 | 0.581 |
|  | rs12258253 | 10 | C/T | 0.25 | 0.14 | 29/180/263 | 35/163/271 | 0.644 | 0.866 | 0.409 |
|  | rs12413745 | 10 | T/C | 0.09 | 0.38 | 4/72/397 | 5/72/394 | 0.928 | 0.971 | 0.594 |
|  | rs1930146 | 10 | C/T | 0.03 | 1 | 0/29/444 | 0/30/441 | 0.534 | 0.524 | - |
|  | rs2384375 | 10 | G/C | 0.28 | 0.57 | 35/194/244 | 40/185/246 | 0.78 | 0.678 | 0.925 |
|  | rs2384437 | 10 | T/C | 0.1 | 0.29 | 6/79/387 | 2/86/382 | 0.803 | 0.488 | 0.124 |
|  | rs2384454 | 10 | T/G | 0.07 | 1 | 2/66/405 | 2/65/404 | 0.608 | 0.596 | 0.939 |
|  | rs4540756 | 10 | T/A | 0.27 | 0.49 | 29/199/244 | 38/181/252 | 0.417 | 0.605 | 0.358 |
|  | rs7095441 | 10 | T/C | 0 | 1 | 0/3/470 | 0/5/466 | 0.443 | 0.436 | - |
|  | rs7097777 | 10 | T/C | 0.18 | 0.34 | 14/142/316 | 11/143/315 | 0.958 | 0.999 | 0.999 |
|  | rs7894926 | 10 | A/G | 0.5 | 0 | 0/472/0 | 0/471/0 | - | - | - |
| PMCA2 | rs34866 | 3 | G/A | 0.29 | 1 | 40/193/240 | 41/196/234 | 0.582 | 0.747 | 0.497 |
|  | rs34918 | 3 | T/C | 0.36 | 0.77 | 65/202/206 | 66/216/189 | 0.152 | 0.125 | 0.479 |
|  | rs35674 | 3 | A/C | 0.01 | 0.07 | 3/8/462 | 1/10/460 | 0.72 | 0.594 | 0.999 |
| POU4F3 | rs891969 | 5 | A/G | 0.22 | 0.19 | 17/166/290 | 19/177/275 | 0.207 | 0.207 | 0.594 |
| RDX | rs4753881 | 11 | C/G | 0.43 | 0.26 | 94/235/144 | 75/242/155 | 0.831 | 0.964 | 0.723 |
| Tecta | rs3781823 | 11 | A/G | 0.5 | 0.36 | 115/224/134 | 118/225/128 | 0.464 | 0.561 | 0.532 |
|  | rs584329 | 11 | A/G | 0.33 | 0.42 | 48/201/223 | 52/222/198 | 0.971 | 0.987 | 0.915 |
|  | rs612969 | 11 | G/A | 0.27 | 0.91 | 34/188/251 | 34/184/256 | 0.402 | 0.682 | 0.221 |
| TMPRSS3 | rs225433 | 21 | G/C | 0.47 | 0.71 | 102/231/140 | 113/231/127 | 0.191 | 0.539 | 0.109 |
| TRPA1 | rs16937976 | 8 | G/C | 0.07 | 0.39 | 0/75/397 | 0/54/417 | 0.27 | 0.285 | - |
|  | rs959976 | 8 | C/T | 0.39 | 0.02 | 70/241/161 | 55/247/170 | 0.354 | 0.506 | 0.382 |
| USH1G (SANS) | rs1013013 | 17 | A/G | 0.23 | 1 | 21/177/275 | 25/167/279 | 0.807 | 0.963 | 0.480 |
| ^*^ additive model adjusted by age, CNE, smoking, drinking; | | | | | | | | | | |
| # dominant model adjusted by age, CNE, smoking, drinking; | | | | | | | | | | |
| § recessive model adjusted by age, CNE, smoking, drinking; | | | | | | | | | | |
| A1:the minor allele; A2: the major allele; | | | | | | | | | | |
| A1A1: homozygous mutation ; A1A2:heterozygous mutation ; A2A2:wild type; | | | | | | | | | | |
